# Finite element analysis and computational fluid dynamics to elucidate the mechanism of distal stent graft-induced new entry after frozen elephant trunk technique

**DOI:** 10.1093/ejcts/ezae392

**Published:** 2024-10-29

**Authors:** Shinri Morodomi, Homare Okamura, Yoshihiro Ujihara, Shukei Sugita, Masanori Nakamura

**Affiliations:** Department of Electrical and Mechanical Engineering, Nagoya Institute of Technology, Aichi, Japan; Department of Cardiovascular Surgery, Saitama Medical Center, Jichi Medical University, Saitama, Japan; Department of Electrical and Mechanical Engineering, Nagoya Institute of Technology, Aichi, Japan; Department of Electrical and Mechanical Engineering, Nagoya Institute of Technology, Aichi, Japan; Department of Electrical and Mechanical Engineering, Nagoya Institute of Technology, Aichi, Japan

**Keywords:** Frozen elephant trunk, Stent graft-induced new entry, Simulation

## Abstract

**OBJECTIVES:**

Distal stent graft-induced new entry (dSINE), a new intimal tear at the distal edge of the frozen elephant trunk (FET), is a complication of FET. Preventive measures for dSINE have not yet been established. This study aimed to clarify the mechanisms underlying the development of dSINE by simulating the mechanical environment at the distal edge of the FET.

**METHODS:**

The stress field in the aortic wall after FET deployment was calculated using finite element analysis. Blood flow in the intraluminal space of the aorta and FET models was simulated using computational fluid dynamics. The simulations were conducted with various oversizing rates of FET ranging from 0 to 30% under the condition of FET with elastic recoil.

**RESULTS:**

The elastic recoil of the FET, which caused its distal edge to push against the greater curvature of the aorta, induced a concentration of circumferential stress and increased wall shear stress (WSS) at the aorta. Elastic recoil also created a discontinuous notch on the lesser curvature of the aorta, causing flow stagnation. An increase in the oversizing rate of the FET widened the large circumferential stress area on the greater curvature and increases the maximum stress. Conversely, a decrease in the oversizing rate of the FET increased the WSS and widened the area with high WSS.

**CONCLUSIONS:**

Circumferential stress concentration due to an oversized FET and high WSS due to an undersized FET can cause a dSINE. The selection of smaller-sized FET alone might not prevent dSINE.

## Graphical abstract

**Figure:**
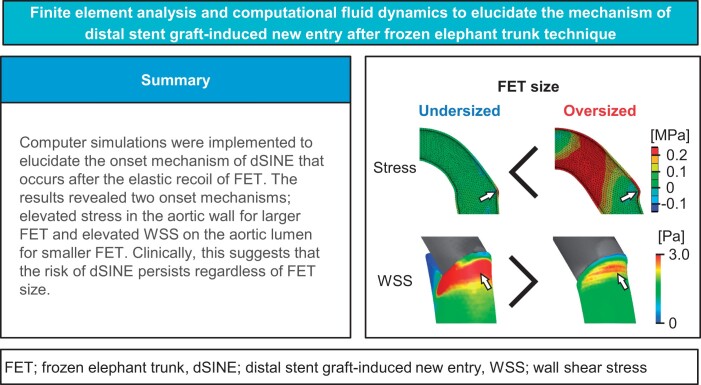

## INTRODUCTION

The frozen elephant trunk (FET) is a hybrid prosthesis consisting of a woven aortic arch graft and an aortic endovascular stent graft. FETs have recently been widely used in aortic dissection surgery. Advantages of FET in acute type A aortic dissection include proximalization of the distal anastomosis, entry tear closure, remodelling of the descending aorta and expansion of the compressed true lumen. The FET provides better postoperative remodelling of the distal aorta than the conventional elephant trunk [1].

Distal stent graft-induced new entry (dSINE) is the development of a new entry tear at the distal end of the FET and is increasingly recognized as a complication after FET or endovascular stent grafting [2]. dSINE reportedly occurs in up to 25% of patients within 3 years of FET implantation [3]. dSINE can result in negative aortic remodelling [4] and rapid diameter expansion [5]. dSINE was reportedly associated with poor late survival when medically managed and can lead to death due to aortic rupture, although not common [6, 7]. However, the mechanisms underlying the development of dSINE remain unclear, and there is no definitive method for preventing dSINE.

Several possible causes of dSINE have been proposed. Oversizing the FET relative to the diameter of the native aorta is believed to provoke a dSINE [8]. However, Hiraoka *et al.* [9] reported that oversizing was not a risk factor for dSINE in their analysis of 177 patients with aortic dissection. As the metallic skeleton of the FET is made of a nitinol alloy, it tends to become straighter over time after implantation. This phenomenon, known as the springback effect or elastic recoil [10], has been reported as a possible cause of dSINE [9]. However, the incidence of dSINE remains relatively high despite careful selection of the size and insertion of the FET. Thus, we hypothesized that there are other mechanisms of dSINE development besides oversizing.

Unphysiological blood flow can trigger vascular diseases. Many studies have demonstrated that long-term imposition of wall shear stress (WSS) at an unphysiological magnitude promotes the activation of cells in the arterial wall, leading to disruption of the internal elastic lamina [11], degeneration of the extracellular matrix [12], arterial inflammation [13] and disruption of the elastic lamina [14, 15]. These studies suggest that insertion and elastic recoil of the FET change haemodynamic conditions and trigger vascular pathological events that lead to dSINE.

Therefore, the present study aimed to explore changes in the mechanical environment after the elastic recoil of an inserted FET, focusing on identifying possible mechanisms of dSINE. Based on the obtained results, we attempted to provide insight into the occurrence of dSINE, regardless of FET size.

## MATERIALS AND METHODS

Because the present study does not deal with any human or animal data and has no ethical problems, we did not obtain any approvals by the Institutional Review Board (IRB) or Ethics Committee (EC) and consent of the patients.

### Structural analysis

**Figure 1:**
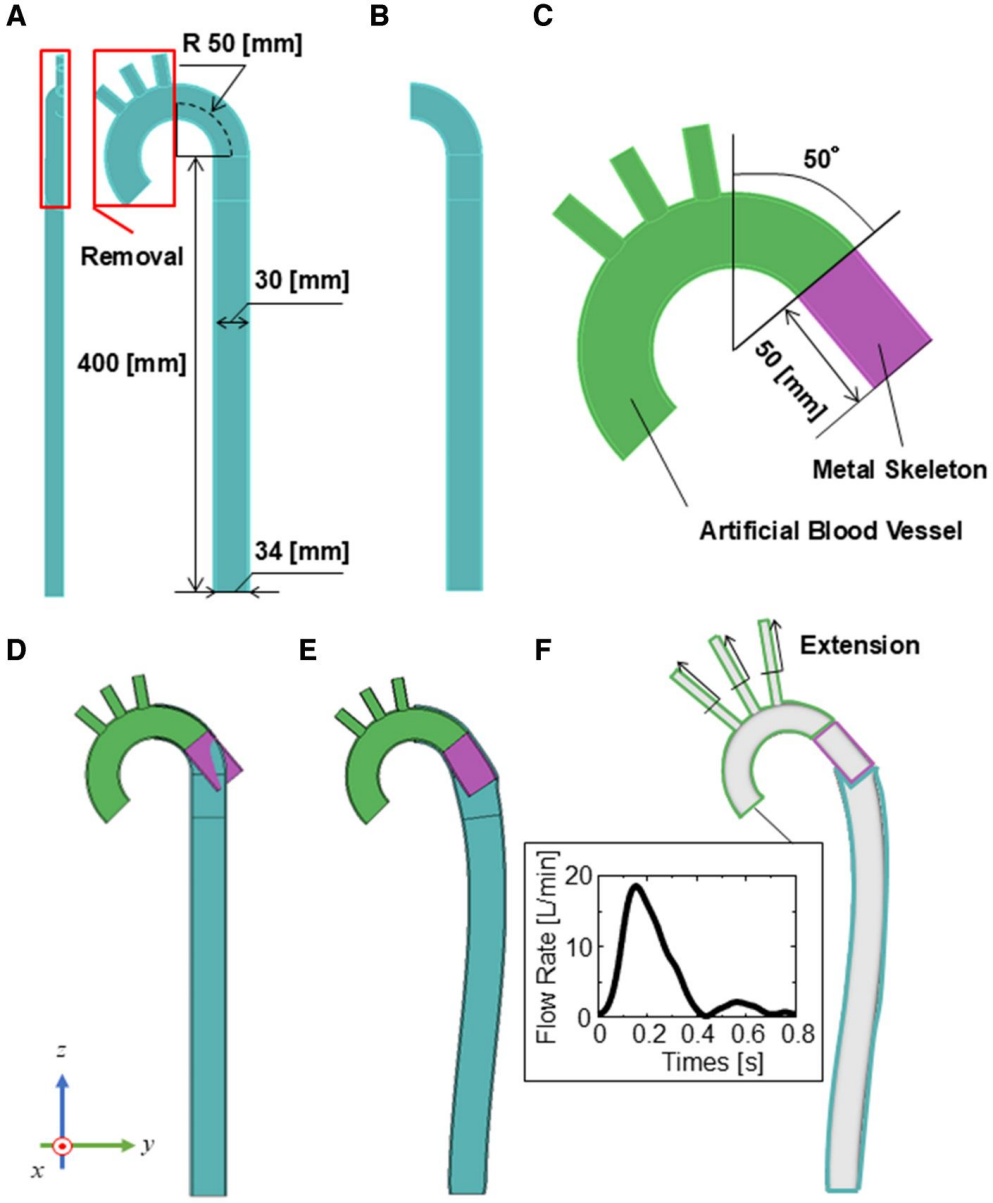
Geometry models for the structural analysis: (**A**) the aorta, (**B**) after removal of the ascending aorta, (**C**) the FET, (**D**) the aorta combined with the FET. (**E**) The geometry obtained after the structural analysis where a physical interaction between the aorta and FET was considered. (**F**) A geometry model of the vascular lumen used for a blood flow simulation. An inlet flow rate waveform provided in the flow simulation is also presented. FET: frozen elephant trunk.

An idealized geometric model of the aorta was created as shown in Fig. 1A. The model was half-symmetrical about the sagittal plane. FET insertion was performed in the aortic model. The section of the aorta squared in red (Fig. 1B) was replaced with an FET model (Fig. 1C), composed of an artificial blood vessel (green) made of polyester and a metallic skeleton covered by polyester (purple). The metallic skeleton was inserted into the remaining aorta to represent the FET treatment (Fig. 1D). The radius of curvature of the ascending aorta and aortic arch was 50 mm, and the length of the descending aorta was 400 mm, with an inner diameter of 30 mm [16] and a wall thickness of 2 mm [17]. The length of the descending aorta was determined such that its downstream end was set as far as possible to minimize the influence of the boundary conditions on the region of interest (distal end of FET). In the natural state, the 50-mm long metallic skeleton is straight, whereas the artificial blood vessel retains its curved shape. Seven FET models with outer diameters of 30–39 mm, corresponding to an oversizing rate of 0–30% at the intervals of 5%, were examined. The thickness of the FET model was set to 1.75 mm based on FET measurements (J Graft FROZENIX, Japan Lifeline, Tokyo, Japan).

Static structural analysis was performed using the ANSYS 2019 R3 software (Cybernet Systems, Tokyo, Japan). The aorta and metal skeletons of the stent graft were modelled as linear elastic materials, while the polyester component of the stent graft was modelled as a rigid body. Young’s modulus was set to 1 MPa for the aorta and 5 MPa for the metal skeleton of the stent graft. The Poisson’s ratio was 0.48 for the metal skeleton and aorta. The downstream end-face of the descending aorta was fixed as the boundary condition. The distal end of the metallic skeleton was forcibly displaced, such that it was completely deployed inside the aortic lumen (Fig. 1E). The elastic recoil of FET was then triggered by releasing the forced displacement of the metallic skeleton part. Structural analysis was conducted under an internal pressure of 100 mmHg, considering the mechanical interaction between the aortic wall and FET by defining “contact” between them. The analysis was conducted when statistical equilibrium was obtained.

### Computational fluid dynamics

A geometric model of the vascular lumen was created from the results of the structural analysis (Fig. 1E). For flow simulations, 3 branches were added to the aortic arches, and the outlets were extended by 50 mm for flow stabilization (Fig. 1F).

Computational fluid dynamics simulations were performed using SCRYU (MSC Software, Tokyo, Japan). Blood was assumed to be an incompressible Newtonian fluid with the viscosity of 0.004 Pa · s and the density of 1050 kg/m^3^. The vessel wall was assumed to be rigid, and nonslip conditions were applied. A physiological flow wave with a heart rate of 75 bpm and a stroke volume of 53 ml was set at the entrance of the FET (Fig. 1F). Three-element Windkessel models representing downstream vasculature and blood flow were established for each outlet. The parameters of the Windkessel models were determined based on Kim *et al.*, [18] with some modifications to ensure blood pressure fell within the physiological range. Blood flow in the 3D domain was numerically coupled to peripheral blood flow models in the 0D domain [19]. The flow simulation was implemented until a cyclically repeatable blood pressure was obtained for all the outlets. WSS, wall shear stress divergence (WSSD) and oscillatory shear index (OSI) [20] were calculated from the flow simulation results. Physically, WSS is the tangential frictional force that occurs at the interface between flowing blood and the vessel wall. OSI measures the degree to which WSS changes direction during 1 cardiac cycle, ranging from 0 (steady-state flow with WSS in 1 direction) to 0.5 (flow with WSS in any direction). WSSD represents how blood flow diverges omnidirectionally, becoming positive and large at the wall with flow impingement.

## RESULTS

### Wall stress and deformation

**Figure 2:**
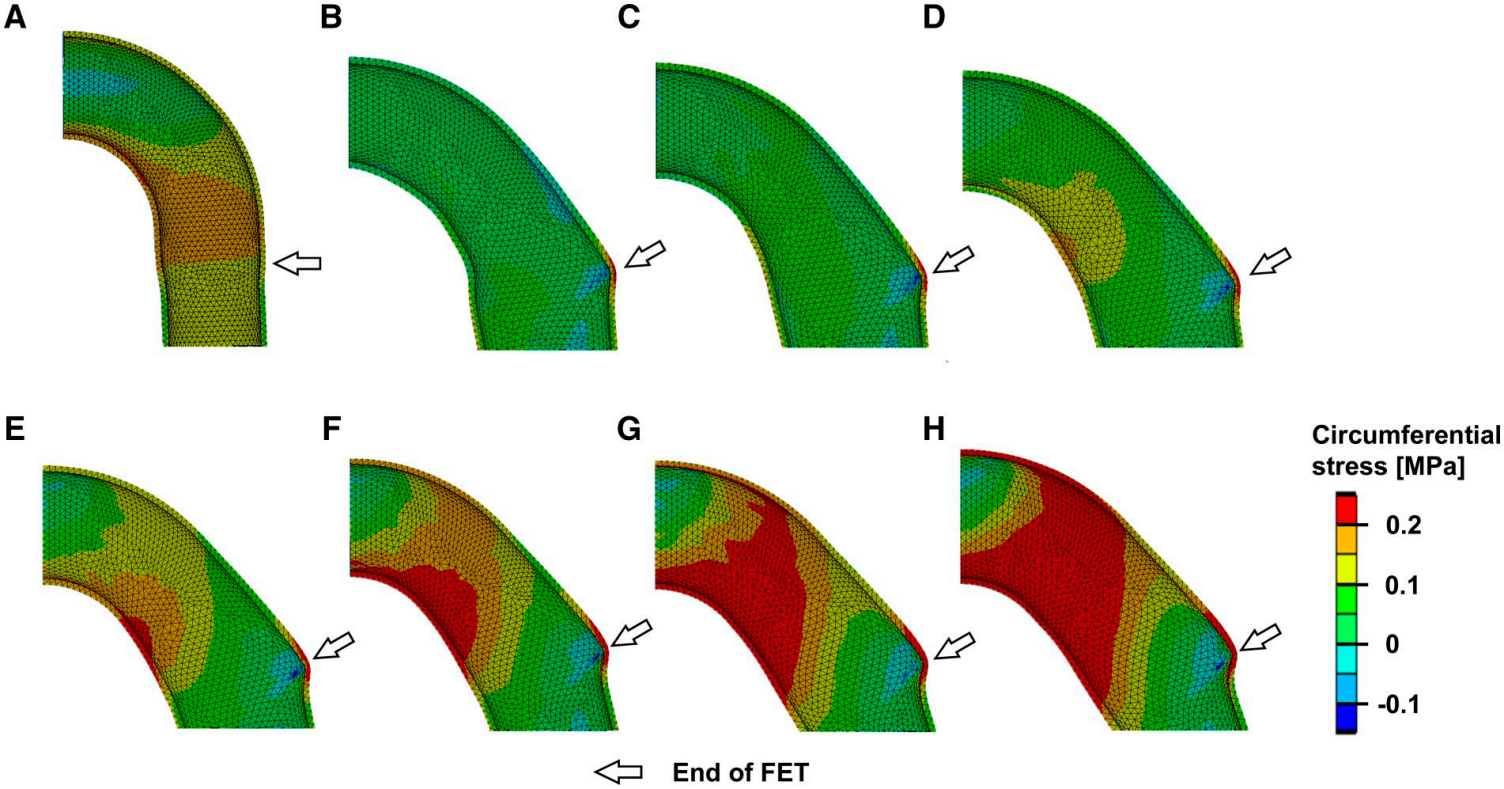
Contour plots of the circumferential stress in the aorta wall (**A**) before (the oversizing rate = 15%) and (**B**–**H**) after the elastic recoil of the FET: (**B**) the oversizing rate = 0%, (**C**) 5%, (**D**) 10%, (**E**) 15%, (**F**) 20%, (**G**) 25% and (**H**) 30%. FET: frozen elephant trunk.

Figure 2 presents the contour plots of circumferential stress in the aortic wall. Before the elastic recoil (immediately after the deployment of the FET), the circumferential stress in the aortic wall was ∼0.1 MPa (Fig. 2A). In contrast, as indicated by the arrows, the elastic recoil caused a stress concentration at the distal edge of the FET, resulting in a circumferential stress exceeding 0.2 MPa. As the oversizing rate increased, the stress concentration intensified, and the region under circumferential stress expanded (Fig. 2B–H).

**Figure 3:**
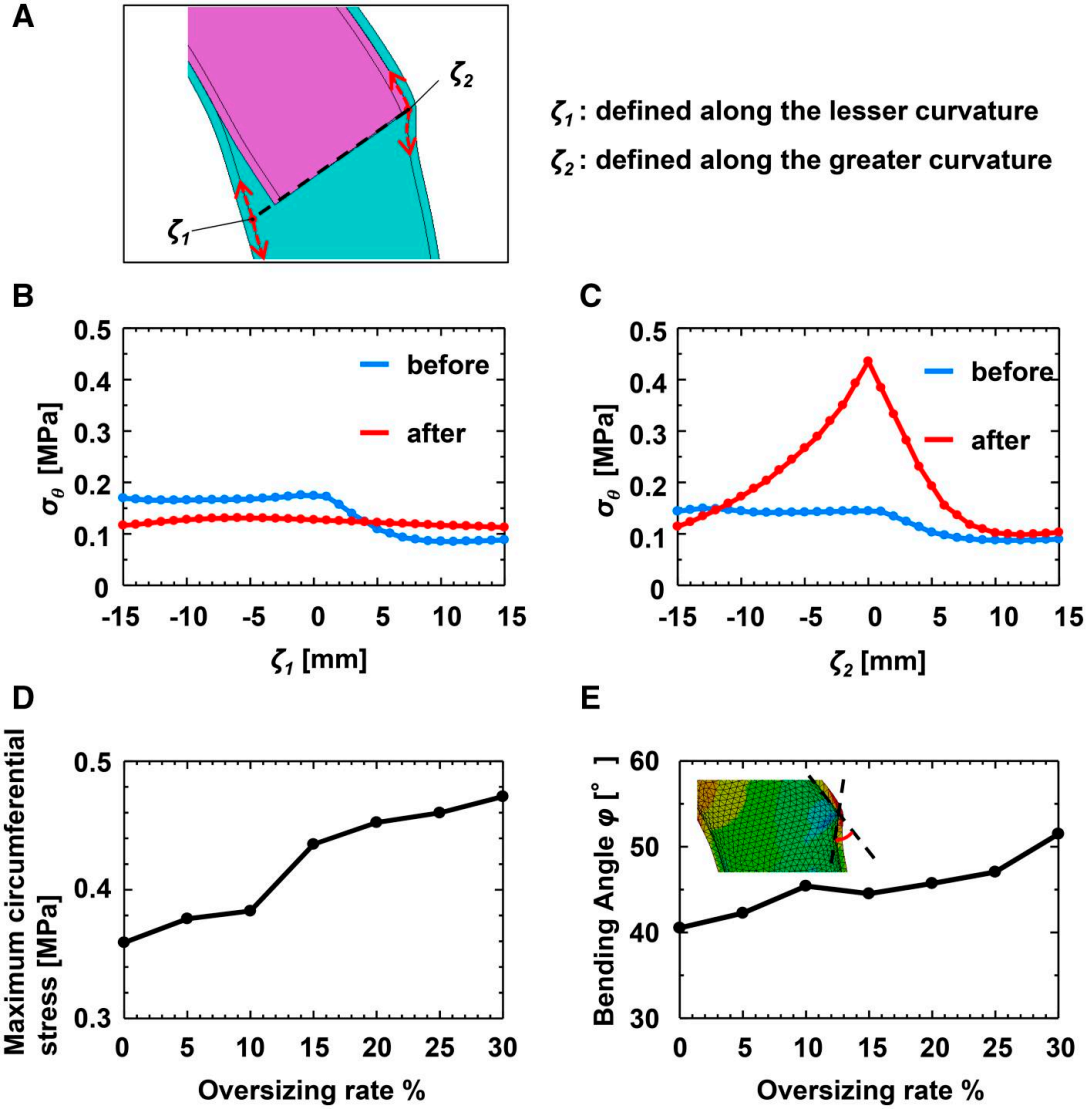
(**A**) Schematic representation of *ζ*_1_ and *ζ*_2_ defined along the lesser curvature and greater curvature of the aorta, respectively. The origin of both path coordinates is at the distal edge of FET. Plots of the circumferential stresses on (**B**) *ζ*_1_ and (**C**) *ζ*_2_ before and after the elastic recoil of the FET in a case of the oversizing rate of 15%. (**D**) Plot of the maximum circumferential stress on *ζ*_2_ after the elastic recoil of FET against the oversizing rate. (**E**) Plot of the bending angle against the oversizing rate. FET: frozen elephant trunk.

Figure 3B and C plot the circumferential stress along path coordinates *ζ*_1_ and *ζ*_2_ (Fig. 3A), defined along the lesser and greater curvature sides of the aortic lumen for an oversizing rate of 15%. As shown in Fig. 3B, there was little change in the circumferential stress on the lesser curvature (path *ζ*_1_) before and after the elastic recoil. In contrast, the circumferential stress on the greater curvature (path *ζ*_2_) showed a marked increase after the elastic recoil, particularly at the distal edge of the FET (*ζ*_2_ = 0 mm) (Fig. 3C). Figure 3D plots the maximum circumferential stress *σ*_max_ over *ζ*_2_ for different oversizing rates, showing higher *σ*_max_ with larger oversizing rate. The bending angle *φ*, defined as the angle between tangential lines of the FET and the descending aorta, increased with higher oversizing rates.

### Blood flow

**Figure 4:**
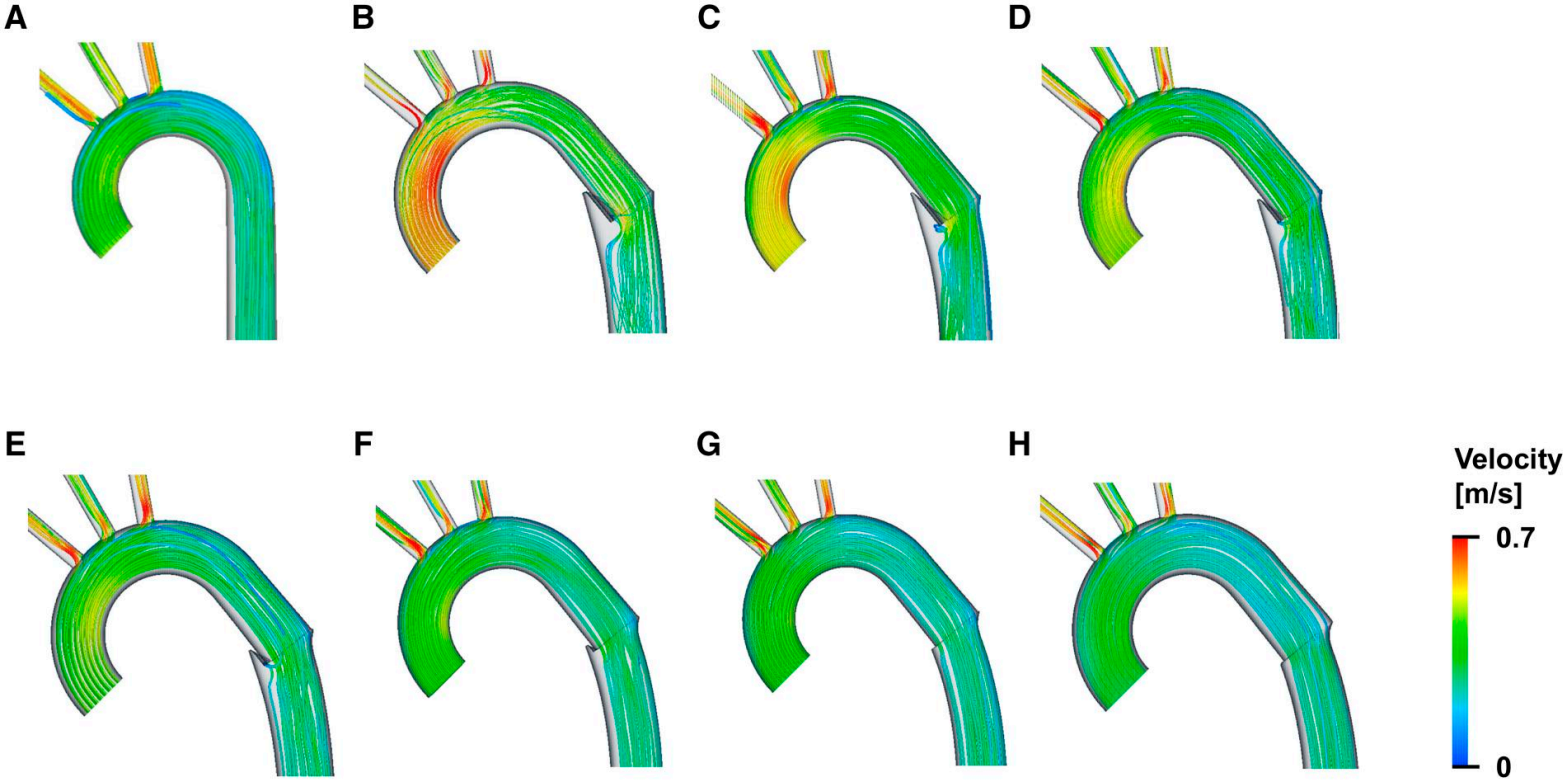
Streamlines obtained (**A**) before the FET treatment, and (**B**–**H**) after the elastic recoil of the FET: (**B**) oversizing rate = 0%, (**C**) 5%, (**D**) 10%, (**E**) 15%, (**F**) 20%, (**G**) 25% and (**H**) 30%. FET: frozen elephant trunk.

Streamlines at the peak systole are shown in Figure 4. The blood flow velocity inside and at the exit of the FET was faster with smaller oversizing rates. A notched area with no streamlines was also observed on the lesser curvature at the distal edge of the FET after elastic recoil. This notched area was larger at a smaller oversizing rate.

**Figure 5:**
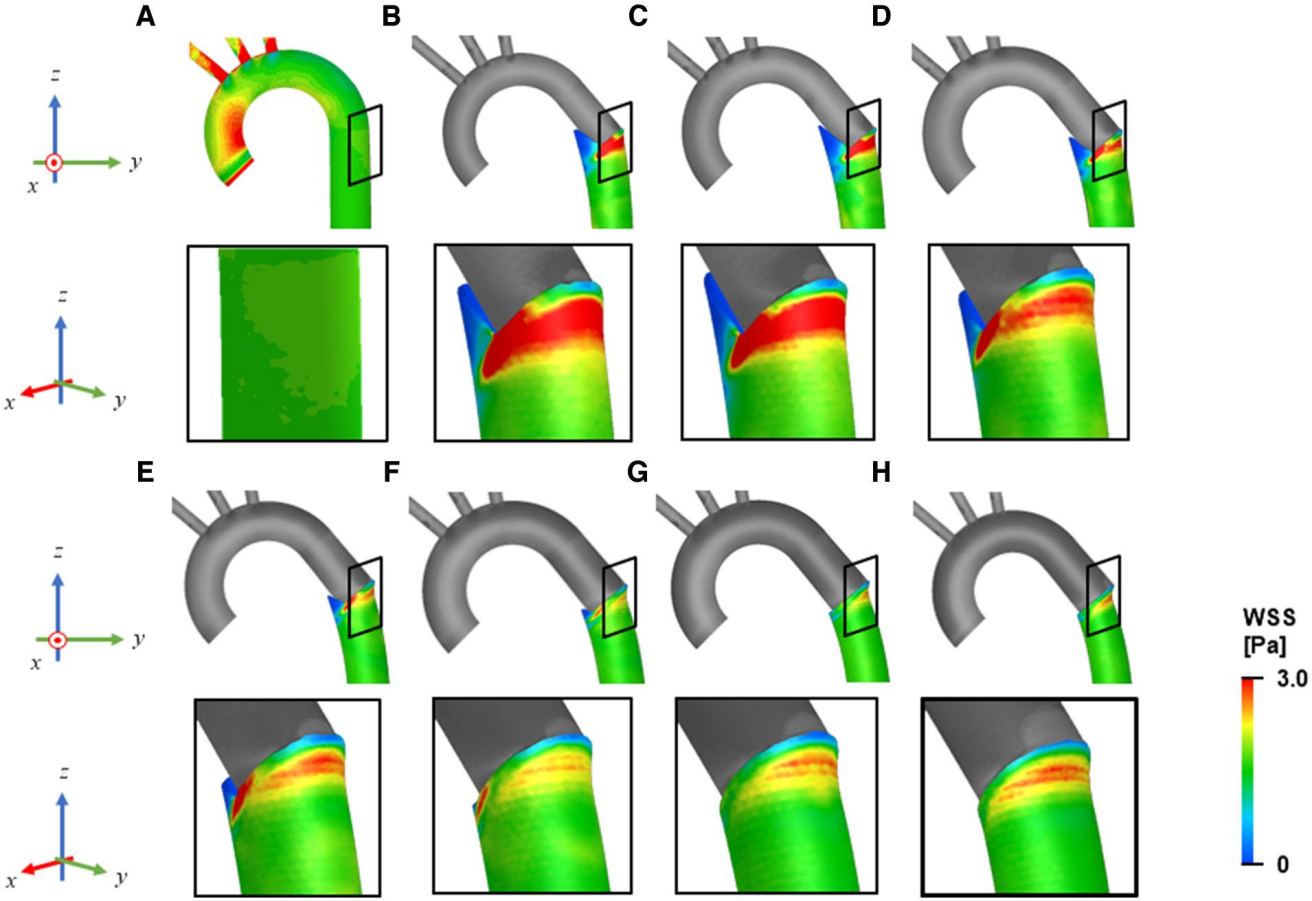
Contour plots of the wall shear stress in the aorta wall (**A**) before the FET treatment, and (**B**–**H**) after the elastic recoil of the FET: (**B**) oversizing rate = 0%, (**C**) 5%, (**D**) 10%, (**E**) 15%, (**F**) 20%, (**G**) 25% and (**H**) 30%. FET: frozen elephant trunk.

Elevation of the WSS was not detected in the descending aorta without FET (Fig. 5A). However, after the deployment of the FET with elastic recoil, a high WSS was noted in the descending aorta (Fig. 5B–H). Particularly, a region with high WSS was locally found on the greater curvature side of the aorta, downstream of the distal edge of the FET. In contrast, the WSS was remarkably low on the luminal surface of the notched area on the lesser curvature.

**Figure 6:**
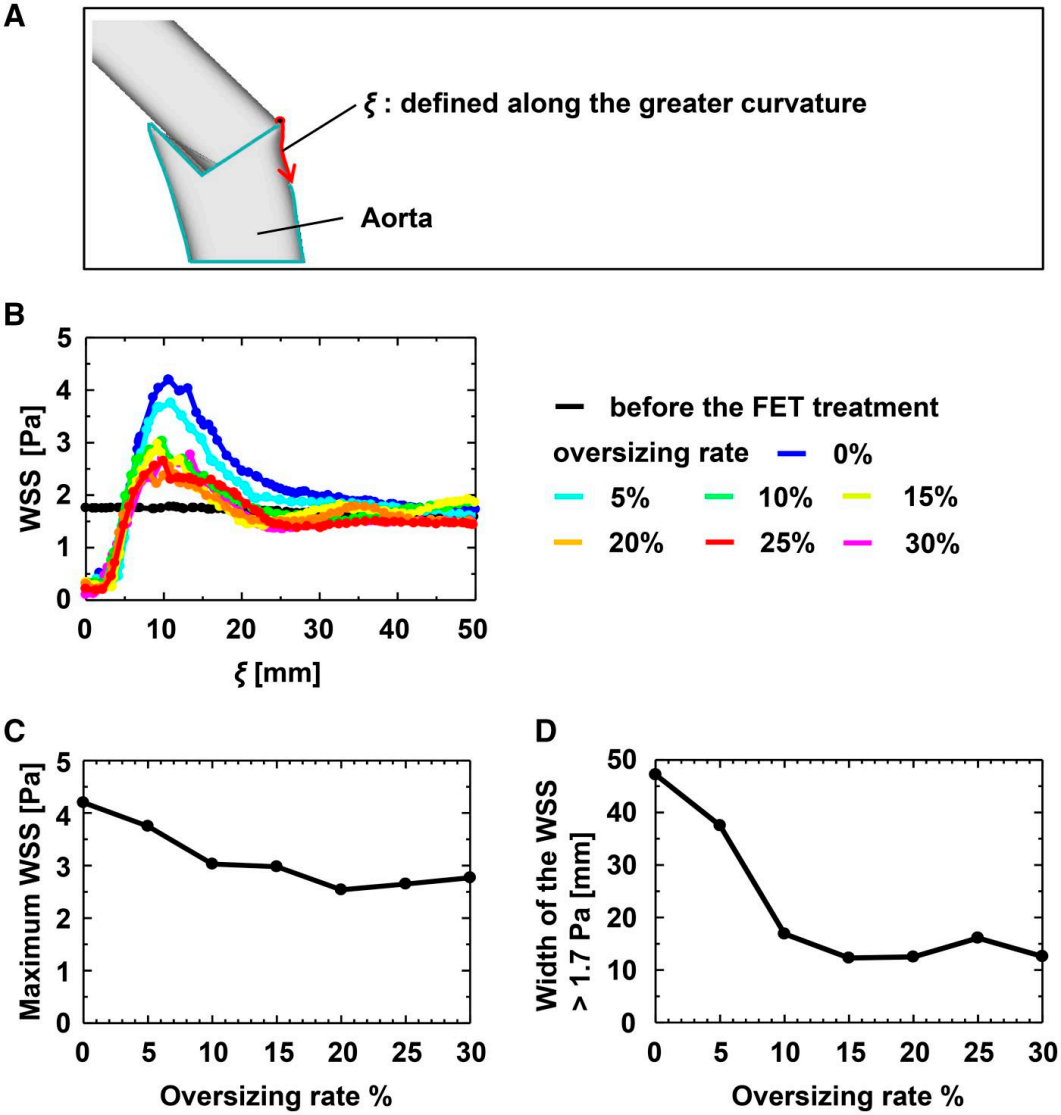
(**A**) Schematic representation of *ξ* defined along the greater curvature of the aorta. (**B**) Plots of the WSS on *ξ* after the elastic recoil of the FET. (**C**) Plot of the maximum WSS on *ξ* after the elastic recoil of FET against the oversizing rate. (**D**) Plot of the length of a part of *ξ* where WSS is >1.7 Pa against the oversizing rate. FET: frozen elephant trunk; WSS: wall shear stress.

WSS along the path coordinate *ξ* from the distal edge of FET is plotted for each oversizing rate (Fig. 6A and B). While WSS along path *ξ* remains nearly constant at ∼1.7 Pa before the deployment of FET (black line), WSS increased after deployment of FET with elastic recoil. The area with the highest WSS was not located at the distal edge of the FET but ∼10 mm distal to the edge of the FET. The WSS was similar to that before the FET deployment in an area 20 mm or more distal to the FET. When comparing the maximum WSS among different oversizing rates, a lower oversizing rate was associated with a higher maximum WSS on the path *ξ* (Fig. 6C). Figure 6D illustrates the relationship between the oversizing rate and areas with high WSS. The path length with a WSS over 1.7 Pa, a reference WSS observed in the aorta without the FET, increased with a lower oversizing rate.

Contour plots of the WSSD and OSI are provided in Supplementary Material, Figs S1 and S2, respectively. A high WSSD was semi-circumferentially observed on the greater curvature side of the aorta just distal to the FET, with the magnitude of the WSSD being higher at lower oversizing rates. In contrast, the OSI was locally high on the lesser curvature at the distal end of the FET, with lower oversizing rates resulting in a higher OSI.

## DISCUSSION

In this study, we implemented finite element analysis to calculate the stresses within the aortic wall, while computational fluid dynamics was used to determine the wall shear stresses at the luminal surface of the aorta after FET deployment. The circumferential stress at the distal end of the FET resulted from the elastic recoil of the device and showed a correlation with the oversizing rate of the FET, increasing as the oversizing rate increased. Computational fluid dynamics further revealed that a smaller-sized FET resulted in faster blood flow velocity and higher WSS at the exit of the FET.

Oversizing the FET relative to the native aorta diameter is commonly recognized as a risk factor for dSINE [8]. However, Hiraoka *et al.* [9] reported that dSINE occurred regardless of the oversizing rate of the FET. As demonstrated in this study, there may be several possible mechanisms contributing to the onset of dSINE. The elastic recoil of the FET induces high circumferential stress and high WSS on the greater curvature of the aortic wall. As expected, the higher the oversizing rate of the FET, the greater and wider is the circumferential stress at the distal end of the FET (Fig. 2). Conversely, the area of high WSS widens with a decrease in the oversizing rate, as shown in Fig. 5. These findings suggest that dSINE can occur regardless of the oversizing rate of the FET. The high circumferential stress and high WSS regions were not necessarily identical; high circumferential stress was detected only at the distal end of the FET (Fig. 2), whereas the maximum WSS region was located ∼10 mm downstream. Therefore, we hypothesize that there are 2 mechanisms for dSINE development in the context of FET sizing.

Elastic recoil, which is the tendency of an FET to straighten over time, is common [9]. Hiraoka *et al.* [9] reported that stronger elastic recoil of the FET was observed in patients with dSINE during follow-up. Even when the distal end of the FET is deployed in the straight portion of the descending aorta, FETs often straighten further and protrude from the aortic wall during follow-up. This deformation can cause significant mechanical stress on the aortic wall. Longer FET might cause less elastic recoil, less WSS and subsequently less dSINE. Clinically, Li *et al.* [6] identified shorter stent graft length as a predictor for dSINE after thoracic endovascular aortic repair. However, regarding FET, some groups showed that there was no association between FET length and incidence of dSINE [9, 21].

The matrix metalloproteinase (MMP) family of enzymes, which mediates protein degradation in the extracellular matrix, is reportedly overexpressed in the vessel wall under high-stress conditions in atherosclerotic lesions [22]. The insertion of an oversized stent graft into porcine aortas results in a decrease in elastin and muscle fibres in the aortic wall [23]. These studies suggest that the overexpression of the MMP family due to high mechanical stress conditions may degrade the extracellular matrix, render the aortic wall vulnerable and consequently lead to the development of dSINE.

Unphysiological haemodynamics induce various vascular cell dysfunctions. Han *et al.* [24] demonstrated that higher WSS increased the production of MMP-2 and MMP-9 by smooth muscle cells in a cell culture model. This increased production of MMPs is expected to result in the degradation of elastin and collagen, which are structural components of the media, making the aortic wall vulnerable and prone to aortic dissection. Wen *et al.* [25] revealed that the location of high WSS coincides with the entry location of the aortic dissection. Moreover, researchers have documented that prolonged exposure to abnormal levels of WSS leads to the degradation of the extracellular matrix [12], arterial inflammation [13] and disruption of the elastic lamina [14, 15]. As shown in Fig. 5, elevated WSS was observed immediately downstream of the distal end of the FET. These studies, combined with the present results, suggest that dSINE was also induced by the elevated WSS that resulted from the deformed aorta, owing to the elastic recoil of the FET.

Misfeld *et al.* [26] reported that early postoperative partial intraluminal thrombosis reportedly occurred in 17% of cases after FET deployment and was associated with a higher in-hospital mortality rate. In that study, thrombosis was more frequently observed in patients with smaller FETs. In our study, a notched area with no streamlines was observed on the lesser curvature of the distal edge of the FET (Fig. 4). It is presumed that the blood flow stagnates in the notch area, leading to thrombus formation. The notched area was larger with a smaller oversizing rate, which may explain the higher thrombosis rate with smaller FETs, as reported by Misfeld *et al.* [26].

Based on our results, the following preventive measures against dSINE are suggested: using a thinner nitinol alloy wire may mitigate the elastic recoil of the FET and lower the radial force of the stent, thereby reducing stress on the aortic wall. Since both oversizing and undersizing of the FET can trigger the development of dSINE, careful selection of the FET size alone may not be effective. Instead, adding a non-stent portion to the distal end of the FET or stent graft with reduced radial force is expected to reduce circumferential stress at the edge of the FET [27]. However, it remains uncertain whether the non-stent portion of the FET can reduce WSS on the aortic wall, as WSS is induced by blood flow and not mechanical stress from the stent itself. Distal anastomosis at zone 3 rather than zone 2 is also expected to mitigate curvature of a deployed FET and might reduce elastic recoil.

### Limitations

This study had some limitations. First, the aortic model was geometrically idealized. The inclusion of more complex geometries, such as torsion [28], might have yielded different stresses and WSS distributions in the aortic model. Future studies should use anatomically realistic geometry created from patient medical images such as CT scans to correlate stress and WSS with clinical results in terms of the presence or absence of dSINE in patient follow-up. Second, the material models used in the structural models may have been oversimplified. Although the aorta is modelled as a linear elastic material, the actual aorta is anisotropic and exhibits a highly nonlinear elastic nature [29]. Flap may have a different material property from a native aorta. Inclusion of the flap would result in a more complex stress field. The polyester part of the FET was modelled as a rigid body since it is firmly sutured to the aorta in the FET procedure. In reality, it is much softer with a Young’s modulus of 1.78 GPa [30], although calculations with the Young’s modulus of 1.78 GPa for the polyester part of FET demonstrated similar results to those obtained with the rigid body (Supplementary Material, Fig. S3). More accurate modelling of FET would also be important as the incidence of dSINE is associated with FET devices used. Third, the development of dSINE should be associated with not only mechanical factors but also patient factors, such as vulnerability of the aortic wall. The higher incidence of dSINE in patients with chronic aortic dissection indicates that patient factors play important roles in the mechanism of dSINE [9]. Although this study focused solely on investigating the mechanical factors, the aetiology of dSINE is considered more complex.

## CONCLUSIONS

The elastic recoil of the FET results in high circumferential stress and high WSS on the aortic wall. High circumferential stress occurs directly beneath the distal edge of the FET, and the stress increases with increasing oversizing rate. Conversely, a high WSS was observed in the aortic lumen slightly downstream from the distal edge of the FET, with its magnitude increasing as the oversizing rate decreased. Therefore, we speculate that the onset mechanism of dSINE involves 2 patterns: the occurrence of high stress on the aortic wall and the imposition of high WSS in the aortic lumen. It is anticipated that the risk of dSINE development persists regardless of whether the FET diameter is large or small relative to the aorta.

## Supplementary Material

ezae392_Supplementary_Data

## Data Availability

All data are available within the article.

## ABBREVIATIONS

dSINE: Distal stent graft–induced new entry
FET: Frozen elephant trunk
MMP: Matrix metalloproteinase
OSI: Oscillatory shear index
WSS: Wall shear stress
WSSD: Wall shear stress divergence
