# Supplementary material for: Finite element analysis and computational fluid dynamics to elucidate the mechanism of distal stent graft-induced new entry after frozen elephant trunk technique
Source: Eur J Cardiothorac Surg. 2024 Oct 29;66(5):ezae392. doi: 10.1093/ejcts/ezae392 (PMC11568347; doi:10.1093/ejcts/ezae392)
Supplement: ezae392_Supplementary_Data [file ezae392_supplementary_data.zip › EJCTS_dSINE_Supplementary Figure_240930_v3.pptx]

## Slide 1
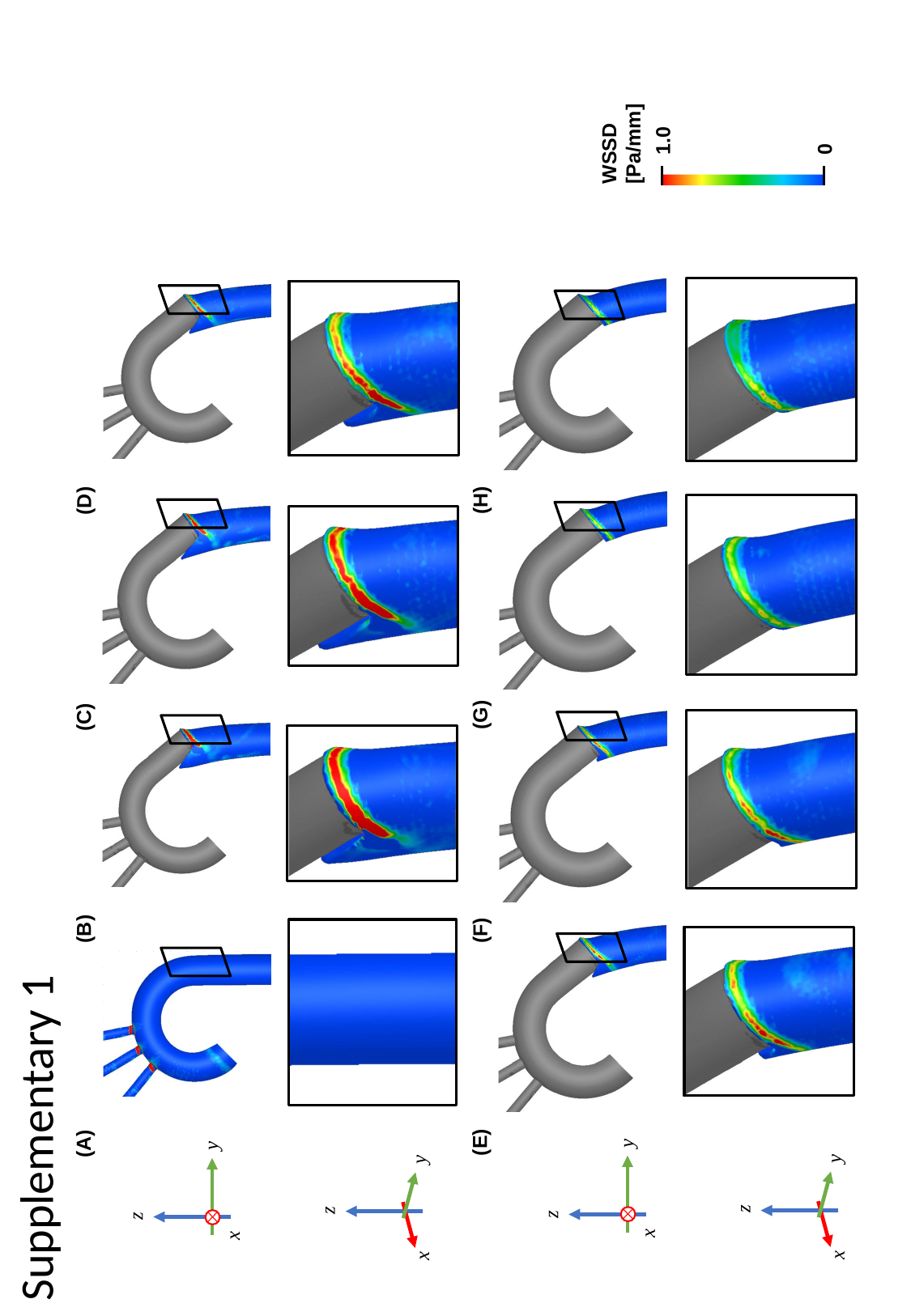

Supplementary 1
(A)
(B)
(C)
(D)
z
y
x
z
y
x
(E)
(F)
(G)
(H)
z
y
x
WSSD
[Pa/mm]
1.0
z
y
x
0

## Slide 2
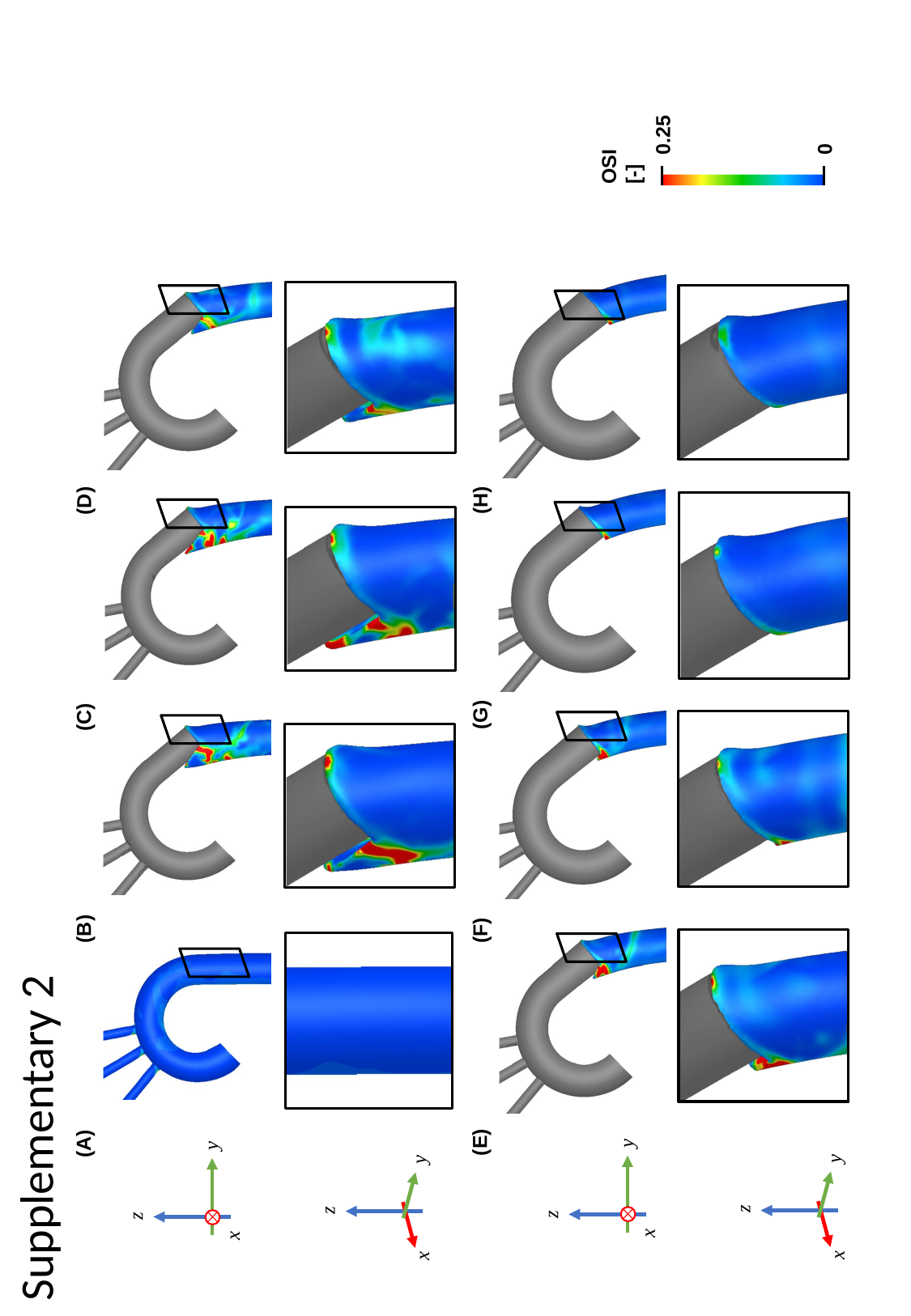

Supplementary 2
(A)
(B)
(C)
(D)
z
y
x
z
y
x
(E)
(F)
(G)
(H)
z
y
x
OSI
[-]
0.25
z
y
x
0

## Slide 3
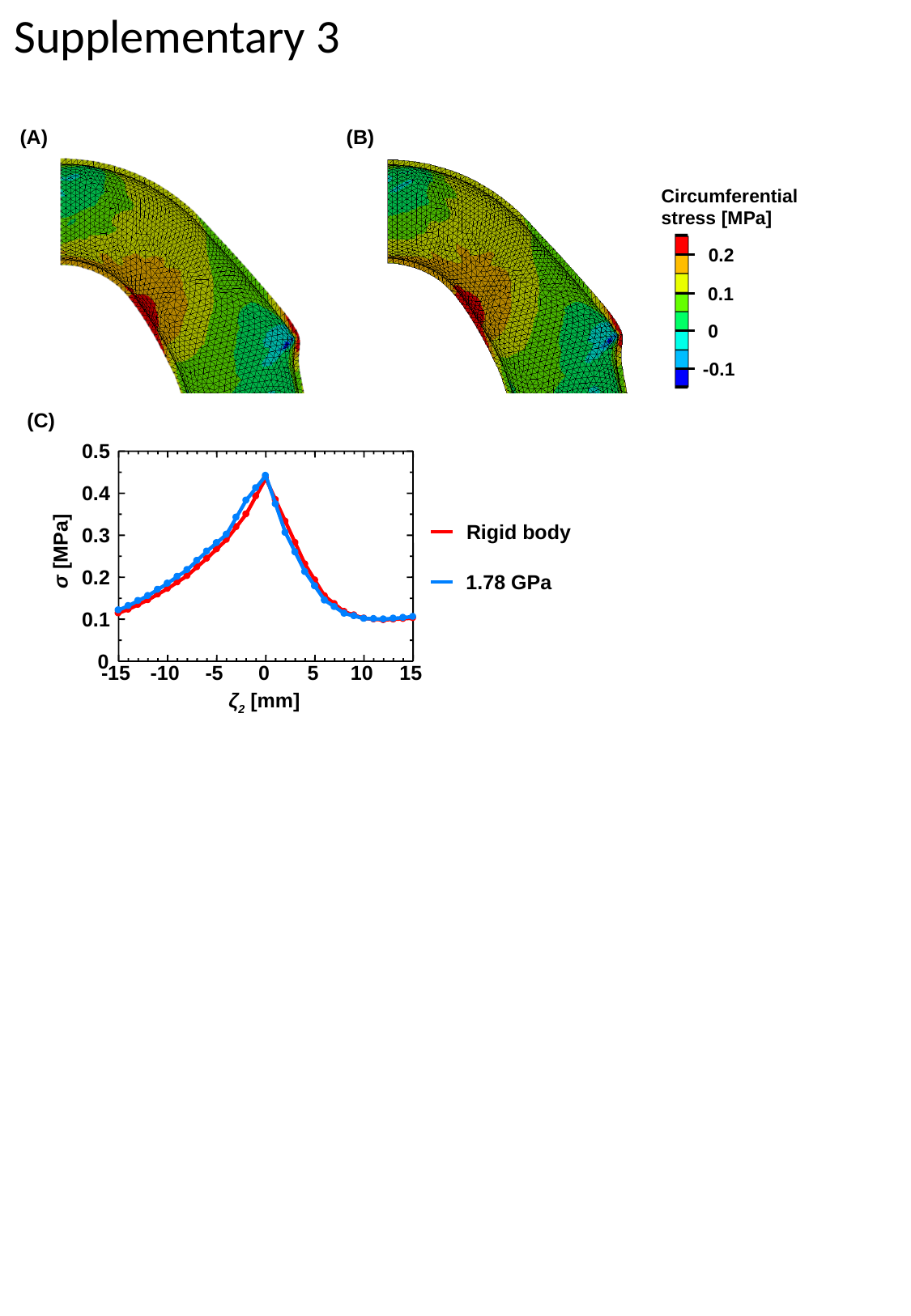

Supplementary 3
(A)
(B)
Circumferential
stress [MPa]
0.2
0
-0.1
0.1
(C)
0.5
0.4
Rigid body
1.78 GPa
0.3
0.2
0.1
0
-15
-10
-5
0
5
10
15
ζ2 [mm]
