# Supplementary material for: Finite element analysis and computational fluid dynamics to elucidate the mechanism of distal stent graft-induced new entry after frozen elephant trunk technique
Source: Eur J Cardiothorac Surg. 2024 Oct 29;66(5):ezae392. doi: 10.1093/ejcts/ezae392 (PMC11568347; doi:10.1093/ejcts/ezae392)
Supplement: ezae392_Supplementary_Data [file ezae392_supplementary_data.zip › Figure_legend_SUPPLEMENTARY MATERIAL_20240930.docx]

**SUPPLEMENTARY MATERIAL, FIGURE S1:** Contour plots of the wall shear stress divergence (WSSD) on the aorta wall (A) before the FET treatment, and (B-H) after the elastic recoil of the FET: (B) oversizing rate = 0%, (C) 5%, (D) 10%, (E) 15%, (F) 20%, (G) 25%, and (H) 30%.

**SUPPLEMENTARY MATERIAL, FIGURE S2:** Contour plots of the oscillatory shear index (OSI) on the aorta wall (A) before the FET treatment, and (B–H) after the elastic recoil of the FET: (B) oversizing rate = 0%, (C) 5%, (D) 10%, (E) 15%, (F) 20%, (G) 25%, and (H) 30%.

**SUPPLEMENTARY MATERIAL, FIGURE S3:** Contour plots of the circumferential stress in the aorta wall after the elastic recoil of the FET (the oversizing rate = 15%): (A) the results with polyester as a rigid body, as in the paper, and (B) with the Young's modulus of polyester set to 1.78 GPa. (C) Plots of the circumferential stresses on ζ_2_ after the elastic recoil.
